# Promoter hypomethylation mediated upregulation of MicroRNA-10b-3p targets FOXO3 to promote the progression of esophageal squamous cell carcinoma (ESCC)

**DOI:** 10.1186/s13046-018-0966-1

**Published:** 2018-12-04

**Authors:** Yi-fang Lu, Jia-rui Yu, Zhao Yang, Guan-xia Zhu, Peng Gao, Huan Wang, Si-yuan Chen, Jie Zhang, Mei-yue Liu, Yi Niu, Xiao-mei Wei, Wei Wang, Feng-jin Ye, Li-xin Zhang, Yue Zhao, Guo-gui Sun

**Affiliations:** 1 Department of medicine, Tangshan gongren Hospital, Tangshan, China; 2 Department of Radiation Oncology, North China University of Science and Technology Affiliated People’s Hospital, Shengli Road, Tangshan, 063000 China; 3 Zhejiang Cancer Research Institute, Zhejiang Cancer Hospital, Hangzhou, 310022 China; 4 Wenzhou Medical College, Wenzhou, China; 5 Department of pathology, North China University of Science and Technology Affiliated People’s Hospital, Tangshan, China

**Keywords:** microRNAs, Esophageal squamous cell carcinoma, Hypomethylation, Biomarker, Therapeutic target

## Abstract

**Background:**

Esophageal cancer is a high incident cancer worldwide with poor survival and limited therapeutic options. Alterations of microRNAs are common in cancers, and many of these micro RNAs are potential therapeutic and diagnostic targets to treat these cancers. miR-10b-3p located in chromosome region 2q31.1, and its expression is frequently increased in esophageal squamous cell carcinoma (ESCC). However, the biological functions, clinical significance and therapeutic implications of miR-10b-3p in ESCC remain unclear.

**Methods:**

The expression levels of miR-10b-3p in ESCC specimens were analyzed by in situ hybridization (ISH) and quantitative reverse transcription polymerase chain reaction (qRT-PCR) assays. Ectopic overexpression of miR-10b-3p in ESCC cells, mouse xenograft model, and metastasis model were used to evaluate the effects of miR-10b-3p on proliferation, and migration of cancer cells. Luciferase reporter assay and Western blot were performed to validate the potential targets of miR-10b-3p after the preliminary screening by computer-aided microarray analysis.

**Results:**

We found that miR-10b-3p expression levels were significantly upregulated in the tumor tissues and serum samples of patients with ESCC. The expression levels of miR-10b-3p in both tumor tissues and serum samples were inversely associated with lymph node metastasis and clinical stages. We identified the expression level of miR-10b-3p in ESCC cancer samples as an independent prognostic marker of the overall survival rates of ESCC patients. We found more frequent hypomethylation of the CpG sites located upstream of the miR-10b-3p gene in the ESCC tissues compared with in the adjacent normal tissues, and the DNA methylation status of miR-10b-3p promoter region inversely correlated with the expression levels of miR-10b-3p. Ectopic overexpression of miR-10b-3p promoted cell proliferation, colony formation, migration and invasion in ESCC. While knockdown of miR-10b-3p had the opposite effects, particularly in promoting apoptosis. Mouse xenograft model confirmed that miR-10b-3p functions as a potent oncogenic miRNA in ESCC, which also promoting ESCC metastasis. Mechanistically, we found miR-10b-3p regulated FOXO3 expression by directly binding to the 3′-untranslated region. And systemic delivery of miR-10b-3p antagomir reduced tumor growth and inhibit FOXO3 protein expression in nude mice.

**Conclusions:**

Collectively, our findings suggested upregulated expression of miR-10b-3p caused by promoter hypomethylation contributed to the progression of ESCC; Thus, miR-10b-3p is a potentially effective biomarker for ESCC that could have further therapeutic implications.

**Electronic supplementary material:**

The online version of this article (10.1186/s13046-018-0966-1) contains supplementary material, which is available to authorized users.

## Background

Esophageal carcinoma is a serious malignancy in terms of both mortality and prognosis [1]. In United States, although only 17,000 new cases of esophageal cancer are diagnosed every year, the estimated death rate is above 80% [2]. In China, esophageal cancer is the 5th leading cause of cancer-related death, claiming nearly one-quarter million lives every year [3]. Esophageal squamous cell carcinoma (ESCC) is the major form of esophageal cancer among Chinese patients [4]. Although diagnostic technologies and therapies have continuously advanced, the overall five-year survival rate is still far from satisfactory [5, 6]. Therefore, it is crucial to identify oncogenes or tumor suppressive genes that can serve as biomarkers for ESCC to develop more efficient therapeutic strategies for ESCC patients.

miRNAs play important roles in the regulation of basic biological processes, such as cell growth, apoptosis and differentiation. They negatively regulate gene expressions at the posttranscriptional level by direct binding to imperfect complementary sites within the 3’UTR of their mRNA targets [7]. In cancer cells miRNAs can act as either oncogenes or tumor suppressors according to their target genes [8]. Several studies have shown that miRNAs could be used as diagnostic and prognostic biomarkers for cancers. For example, miR-195 expression was shown to be lower in ESCC tissues and to be associated with poor survival outcome [9]. In colorectal cancer, high levels of miR-135b expression and low levels of miR-590-5p expression are associated with clinical stages and survival progression [10, 11].

The coding gene of miR-10b is located in chromosome region 2q31.1, which has one of the largest miRNA clusters [12–16]. The mammalian miR-10b family includes miR-10b-3p and miR-10b-5p. Although miR-10b-3p and miR-10b-5p have identical seed sequences, they probably regulate different pathways. Recent research has demonstrated that the expression of miR-10b-3p in serum maybe used as a biomarker for the diagnosis of hepatocellular carcinoma (HCC), and in the prediction of survival in patients treated with sorafenib by its association with macrovascular invasion (MVI) [17]. However, the exact biological functions and regulatory mechanisms of miR-10b-3p in human cancer are largely unknown.

In the present study, we set up to examine the expression profiles and prognostic value of miR-10b-3p in ESCC. We identified serum miR-10b-3p as a noninvasive biomarker for ESCC. In addition, we identified FOXO3 as an important downstream target for miR-10b-3p and the underlying molecular mechanisms for miR-10b-3p overexpression to promote ESCC progression. By using ESCC mouse xenograft model, we found miR-10b-3p promotes multiple aspects of tumor development, including tumor growth and metastasis, and we verified the inversed correlation between miR-10b-3p and FOXO3 by IHC assays with the ESCC mouse xenograft model and human ESCC tissue samples.

## Materials and methods

### Tissue samples and ethics statement

An organized chip array including 93 ESCC tissues and nonneoplastic esophageal tissues was purchased from Outdo Biotech (HEso-Squ180Sur-02 and HEso-Squ180Sur-03, Shanghai, China; http://www.superchip.com.cn/, Table 1). Another 102 paired frozen paraffin ESCC tissues and matched adjacent noncancerous tissues were obtained from North China University of Science and Technology Affiliated People’s Hospital from 2009 to 2013 (Table 2). Serum samples from 92 ESCC patients and 52 healthy controls were obtained from the above mentioned hospital (Table 3). Another 103 paired frozen paraffin ESCC tissues and matched adjacent noncancerous tissues were obtained from North China University of Science and Technology Affiliated People’s Hospital from 2013 to 2016 (Table 4). All serum specimens were transported at 4 °C and stored at − 80 °C until RNA extraction. This study was carried out after obtaining approval from the ethics committee of the hospital and informed consent from all subjects. All patient samples were obtained with full written consent, and all samples were collected from the remained tissues after the completion of pathological diagnosis.

**Table 1:** Correlations between miR-10b-3p expression and clinicopathological parameters of ESCC patients

Characteristics	Training group (*n* = 93)			Test group (*n* = 102)		
	Low (*n* = 21)	High (*n* = 72)	*P*	Low (*n* = 27)	High (*n* = 75)	*P*
Gender						
Male	17 (22.1%)	60 (77.9%)	0.799	21 (25.0%)	63 (75.0%)	0.467
Female	4 (25.0%)	12 (75.0%)		6 (33.3%)	12 (66.7%)	
Age						
≤ 60	6 (22.2%)	21 (77.8%)	0.958	8 (22.9%)	27 (77.1%)	0.550
>60	15 (22.7%)	51 (77.3%)		19 (28.4%)	48 (71.6%)	
Tumor size						
< 5 cm	10 (19.8%)	41 (80.4%)	0.450	20 (30.3%)	46 (69.7%)	0.235
≥ 5 cm	11 (26.2%)	31 (73.8%)		7 (19.4%)	29 (80.6%)	
Tumor stage^a^						
T1+ T2	7 (31.8%)	15 (68.2%)	0.270	9 (39.1%)	14 (60.9%)	0.118
T3+ T4	13 (20.3%)	51 (79.7%)		18 (22.8%)	61 (77.2%)	
Histological grade						
Well/moderate	17 (23.0%)	57 (77.0%)	0.858	21 (25.3%)	62 (74.7%)	0.576
Poor/NS	4 (21.1%)	15 (78.9%)		6 (31.6%)	13 (68.4%)	
Lymph node metastasis						
Negative	17 (37.8%)	28 (62.2%)	0.001	20 (33.9%)	39 (66.1%)	0.046
Positive	4 (8.3%)	44 (91.7%)		7 (16.3%)	36 (83.7%)	
Clinical stages^a^						
I + II	17 (35.4%)	31 (64.6%)	0.002	22 (37.9%)	36 (62.1%)	0.003
III + IV	3 (7.5%)	37 (92.5%)		5 (11.4%)	39 (88.6%)	

^a^Numbers do not equal to the total number due to missing data

**Table 2:** Multivariate cox regression analysis of factors associated with OS in ESCC

Variable	Training group (*n* = 93)			Test group (*n* = 102)		
	95% CI	*RR*	*P*	95% CI	*RR*	*P*
Sex (male vs. female)	0.194–1.405	0.522	0.198	0.268–1.214	0.570	0.145
Age (≤60 vs. > 60 years)	0.796–2.877	1.514	0.206	0.573–1.674	0.980	0.940
Tumor size (≤5 cm vs. > 5 cm)	0.667–2.119	1.189	0.558	0.396–1.229	0.698	0.213
Tumor stages (T1 + T2 vs. T3+ T4)	0.643–3.247	1.445	0.373	0.555–2.548	1.189	0.656
Histologic grade (Well/ moderate vs. Poor/NS)	0.880–3.517	1.759	0.110	0.491–1.852	0.954	0.889
Lymph node metastasis (negative vs. positive)	0.109–4.526	0.702	0.709	0.306–1.951	0.773	0.586
Clinical stages (I + II vs. III + IV)	0.297–12.497	12.806	0.487	0.616–4.530	1.670	0.314
miR-10b-3p expression levels (Low vs. High)	1.355–7.062	3.093	0.007	1.678–7.913	3.644	0.001

**Table 3:** The serum of miR-10b-3p expression status and clinicopathological characteristics of patients with ESCC

Characteristics	miR-10b-3p expression status		
	Low (*n* = 46)	High (*n* = 46)	*P*
Gender			
Male	33 (47.8%)	36 (52.2%)	0.470
Female	13 (56.5%)	10 (43.5%)	
Age			
<60	19 (44.2%)	24 (55.8%)	0.296
≥ 60	27 (55.1%)	22 (44.9%)	
Tumor size			
< 5 cm	31 (51.7%)	29 (77.5%)	0.662
≥ 5 cm	15 (22.5%)	17 (22.5%)	
Tumor stage			
T1+ T2	18 (60.0%)	12 (40.0%)	0.182
T3+ T4	28 (45.2%)	34 (54.8%)	
Histological grade			
Well/moderate	36 (53.7%)	31 (46.3%)	0.241
Poor/NS	10 (40.0%)	15 (60.0%)	
Lymph node metastasis			
Negative	26 (65.0%)	14 (35.0%)	0.012
Positive	20 (38.5%)	32 (61.5%)	
Clinical stages			
I + II	37 (69.8%)	16 (30.2%)	0.000
III + IV	9 (23.1%)	30 (76.9%)

**Table 4:** Correlation between FOXO3 expression and clinicopathological parameters of ESCC patients

Characteristics	FOXO3 expression status		
	Low (*n* = 71)	High (*n* = 32)	*P*
Gender			
Male	60 (70.6%)	25 (29.4%)	0.430
Female	11 (61.1%)	7 (38.9%)	
Age			
<60	25 (71.4%)	10 (28.6%)	0.700
≥ 60	44 (67.7%)	21 (32.3%)	
Tumor size			
< 5 cm	41 (69.5%)	18 (30.5%)	0.899
≥ 5 cm	28 (68.3%)	13 (31.6%)	
Tumor stage			
T1+ T2	7 (58.3%)	5 (41.7%)	0.399
T3+ T4	64 (70.3%)	27 (29.7%)	
Histological grade			
Well/moderate	51 (68.0%)	24 (32.0%)	0.738
Poor/NS	20 (68.9%)	8 (28.6%)	
Lymph node metastasis^a^			
Negative	26 (56.5%)	20 (43.5%)	0.011
Positive	44 (80.0%)	11 (20.0%)	
Clinical stages^a^			
I + II	22 (56.4%)	17 (43.6%)	0.026
III + IV	48 (69.3%)	14 (30.7%)	

^a^Numbers do not equal to the total number due to missing data

### Cell lines and cell culture

The human ESCC cell lines TE-1, EC-109, KYSE30, KYSE150, KYSE180, KYSE450 and KYSE510 were obtained from the Cell Culture Center of Peking Union Medical College (Beijing, China) and Typical Culture Cell Bank of Chinese Academy of Sciences (Shanghai, China). The human embryonic kidney (HEK) 293 T cell line was obtained from ATCC (Manassas, VA). Human ESCC cell lines were cultured in RPMI-1640 medium, and HEK 293 T cells were maintained in DMEM supplemented with 10% fetal bovine serum (Gibco BRL, Grand Island, NY) in a humidified atmosphere of 5% CO_2_ at 37 °C.

### In situ hybridization of miR-10b-3p

The miR-10b-3p probe was tagged with 3′ and 5′ digoxigenin and modified with LNA nucleotides (Redlandbio.biomart.cn, Guangzhou, China). The miR-10b-3p probe (*5′- TTCCCCTAGAATCGAATCTGT-3′*) was tagged with 3′ and 5′ digoxigenin (Redlandbio.biomart.cn, Guangzhou, China). The probe-target complex was detected using an anti-digoxigenin-alkaline phosphate conjugate, nitroblue tetrazolium and 5-bromo-4-chloro-3′-indolyphosphate as the chromogen. As for detection of miR-10b-3p in situ, specimens were incubated with proteinase K (15 μg/ml) under 37 °C for 10 min. The specimens were washed with PBS and dehydrated using sequentially increased concentrations of ethanol. miR-10b-3p probe was added on specimens and ensuing incubation was performed under 60 °C for 1 h. When incubation ends, we washed specimens briefly in pre-warmed 5×, 1× and 0.2× SSC (60 °C) in sequence. The primary antibody against DIG (1:800) was incubated with specimens under room temperature for 60 min, and the substrate NBT/BCIP was added and incubated in dark for about 15 min. When specific blue signal was observed and KTBT was used to stop further reaction. All procedures were performed under RNase-free condition. Samples were classified according to cytoplasmic miR-10b-3p intensity as follows: negative = negative or faint expression in most cells, low expression = low expression in most cells or moderate expression in < 50% of the cells, and high expression = moderate to strong expression in most cells.

### DNA extraction and bisulfite modification

Genomic DNA was prepared from ESCC cell lines and 18 pairs of freshly frozen ESCC tissues and matched adjacent noncancerous tissues. Purified genomic bisulfite-converted DNA samples were also successfully tested by PCR with the human miR-10b-3p primers 5′-aggaagagagGATTTTGGTAGAAGAATGAGGGAAT-3′ (forward), 5′-cagtaatacgactcactatagggagaaggctTCTTTTCAACACCCAAAAAATACTC-3′ (reverse) to show that the samples could be used for follow-up experiments. A NanoDrop 2000 spectrophotometer was used to measure the converted DNA (Thermo). Then, transformed DNA was PCR-amplified using a TaKaRa rTaq Kit.

### Quantitative analysis of DNA methylation

The UCSC genome browser (https://genome.ucsc.edu/) was used to identify the sequence of the CpG sites. Primer sets for the methylation analysis of the miR-10b-3p promoter were designed using EpiDesigner (https://www.epidesigner.com/start3.html). For each reverse primer, an additional T7 promoter tag was added for in vivo transcription, and a 10-mer tag was added to the forward primer to adjust for the melting temperature. Methylation of miR-10b-3p was quantitatively analyzed by the MassARRAY platform (Agena Bioscience, Inc.). Matrix-assisted laser desorption/ionization time-of-flight mass spectrometry (MALDI-TOF MS), a new type of high-throughput quantitative methylation detection method, was combined with the base specificity of the enzyme reaction to test the DNA methylation level. Mass spectra were collected by MassARRAY Compact MALDI-TOF (Agena Bioscience, Inc.), and the methylation proportions of individual units were generated by EpiTyper 1.0.5 (Agena Bioscience, Inc.). Nonapplicable readings and their corresponding sites were eliminated from analysis. The methylation level was expressed as the percentage of methylated cytosines over the total number of methylated and unmethylated cytosines.

### Cell line treatment with an epigenetic-modulating drug

The ESCC cell lines were treated with 1.5 mmol/L 5-Aza-20-deoxycytidine (Sigma A3656) for 96 h. Twenty-four hours before harvest, 0.5 mmol/L trichostatin A (Sigma T8552) was added. DNA, RNA, and protein were extracted and analyzed for the methylation status of the miR-10b-3p promoter as well as the expression of miR-10b-3p and its target proteins.

### miRNA transfection

All endogenous mature miRNA mimics, inhibitors and agomirs were purchased from RiboBio (Guangzhou, China). For transfection, experimental protocols were performed according to the manufacturer’s protocols. miRNA mimics, miRNA inhibitors and miRNA NC were transfected into cells using Lipofectamine 2000 (Invitrogen, Carlsbad, USA) according to the manufacturer’s instructions. After 48 h of transfection, cells were used for further experiments.

### Plasmid construction

pDonR223-FOXO3 plasmids carrying the human FOXO3 gene were purchased from Changsha Axybio Bio-Tech Co., Ltd. (Changsha, China). The complete coding sequences of human FOXO3 were amplified from pDonR223-FOXO3 plasmids. FOXO3 products and pEGFP-N1 plasmid were digested with Xho I and Hind III; fragments were purified and ligated with T4 DNA ligase. The ligated product was transformed into TOP10 competent cells, and the positive clone was named pEGFP-N1-FOXO3.

### Quantitative real-time polymerase chain reaction

To evaluate the expression of miR-10b-3p and FOXO3, total RNAs were used for the reverse transcription (RT) reactions, and quantitative polymerase chain reaction (qRT-PCR) was performed on a StepOnePlus Real-Time PCR System (AB Applied Biosystems, Carlsbad, CA). U6 and GAPDH were used as internal controls.

### Target prediction and luciferase reporter assays

Bioinformatics analysis was performed using the following programs: miRWalk, miRDB and miRTarBase. The 3′-untranslated region (3’UTR) of human FOXO3 was amplified from human genomic DNA and individually inserted into the pmiR-RB-REPORT vector (Ribobio, Guangzhou, China) using the Xho I and Not I sites. Similarly, the fragment of the FOXO3 3’UTR mutant was inserted into the pmiR-RB-REPORT control vector at the same sites. For reporter assays, ESCC cells were cotransfected with wild-type reporter plasmid and miR-10b-3p mimics. Firefly and Renilla luciferase activities were measured in cell lysates using the Dual-Luciferase Reporter Assay System. Luciferase activity was measured forty-eight hours posttransfection using the Dual-Glo Luciferase Reporter System according to the manufacturer’s instructions. Firefly luciferase units were normalized against Renilla luciferase units to control for transfection efficiency.

### In vitro cell proliferation assays

For cell proliferation assays, cells were seeded into each well of a 96-well plate (5× 103 per well), and the cell proliferation ability was determined by MTS (3-(4,5-dimethylthiazol-2-yl)-5-(3–carboxymethoxyphenyl)-2-(4-sulfophenyl)-2H- tetrazolium) according to the manufacturer’s instructions. MTS solution was added (20 μl/well) to each well and incubated at 37 °C for 2 h. The optical density of each sample was immediately measured using a microplate reader (BioRad, Hercules, CA, USA) at 570 nm.

### Colony formation assay

ESCC cells were transfected with miR-10b-3p mimic or with miR mimic NC, miR-10b-3p inhibitor or miR inhibitor NC. Twenty-four hours later, transfected cells were trypsinized, counted and replated at a density of 1 × 10^3^ cells/10 cm dish. Ten days later, colonies resulting from the surviving cells were fixed with 3.7% methanol, stained with 0.1% crystal violet and counted. Colonies containing at least 50 cells were scored. Each assay was performed in triplicate.

### Transwell migration/invasion assay

In vitro cell migration assays were performed according to the manufacturer’s instructions using transwell chambers (8 μM pore size; Costar). Cells were allowed to grow to subconfluency (~ 75–80%) and were serum starved for 24 h. After detachment with trypsin, cells were washed with PBS and resuspended in serum-free medium. Next, a 100 μl cell suspension (5× 10^4^ cells/mL) was added to the upper chamber. Complete medium was added to the bottom wells of the chambers. For the screen, the cells that had not migrated after 24 h were removed from the upper face of the filters using cotton swabs, but the cells that had migrated were fixed with 5% glutaraldehyde solution to determine the number of migratory cells. The lower surfaces of the filters were stained with 0.25% Trypan Blue. Images of six different × 10 fields from each membrane were acquired, and the number of migratory cells was counted. The mean of triplicate assays for each experimental condition was used. Similar inserts coated with Matrigel were used to evaluate the cell invasive potential in the invasion assay.

### Flow cytometric analysis

Fluorescence-activated cell sorting (FACS) analysis was performed 48 h posttransfection. The cells were harvested, washed with cold PBS, fixed in 70% ethanol at − 20 °C for 24 h, stained with 50 μg/mL propidium iodide (PI) (4ABio, China), and analyzed using a FACSCalibur flow cytometer (BD Biosciences, MA). The results were analyzed using ModFit software (BD Biosciences, USA). Three independent assays were conducted.

### Western blot analysis

For western blot analyses, RIPA buffer containing protease inhibitors and phosphatase inhibitors (Roche) was used to prepare whole-cell lysates. Briefly, equal amounts of lysate were separated by SDS-polyacrylamide gel electrophoresis (SDS-PAGE) and then transferred to PVDF membranes (Millipore). After blocking the membranes with 5% bovine serum albumin (BSA), they were probed with anti-FOXO3 and anti-GAPDH (ab12162, ab8425, Abcam, Cambridge, UK), followed by incubation with the horseradish peroxidase–conjugated secondary antibodies goat-anti-mouse IgG (1:2000) and goat-anti-rabbit IgG (1:3000). Proteins were visualized by Image Reader LAS-4000 (Fujifilm) and analyzed by Multi Gauge V3.2 software.

### Generation of stable cell lines

Recombinant lentiviral vectors for miR-10b-3p overexpression and irrelevant sequences were purchased from XIEBHC Biotechnology (Beijing, China). In addition to the lentivirus expression vectors, there was a luciferase and puromycin reporter gene driven by the EF1α promoter to indicate the infection efficiency in a timely manner. To construct lentiviral vectors, the precursor sequence for miR-10b-3p and the irrelevant sequence (negative control) were inserted into pHBLV-U6-MCS- EF1α-Luc-T2A-puromycin lentiviral vectors. The recombinant lentiviruses were packaged by cotransfection of HEK 293 T cells with pSPAX2 and pMD2.G with LipoFiter reagent. The supernatants with lentivirus particles were harvested at 48 h and 72 h after transfection and filtered through 0.45-μm cellulose acetate filters (Millipore, USA). Recombinant lentiviruses were concentrated by ultracentrifugation. To establish stable cell lines, ESCC cells were transduced with lentivirus with an MOI of approximately 5 in the presence of 5 μg/mL polybrene. The supernatant was removed after 24 h and replaced with fresh complete culture medium. Infection efficiency was confirmed by RT-PCR 96 h after infection, and the cells were selected with 2 μg/ml puromycin for 2 weeks.

### Tumorigenicity and metastasis assays in vivo

All animals received humane care in compliance with the “Guide for the Care and Use of Laboratory Animals” prepared by the Institute of Laboratory Animal Resources published by the National Institutes of Health and according to the Animal Experiment Guidelines of Samsung Biomedical Research Institute. The effect of miR-10b-3p on the tumorigenic and metastatic potential of ESCC cells was analyzed in subcutaneous and systemic metastasis in vivo models by right subcutaneous tissue and tail vein injection, respectively. For the subcutaneous model, 4–6-week-old BALB/c nude mice were injected subcutaneously in the right hip with 1 × 10^6^ transfected cells. For the experimental metastasis in vivo model, transfected cancer cells (1 × 10^6^ in 100 μL of HBSS) were directly injected into the tail vein. Five weeks later, tumor colonies in subcutaneous tissue were observed by HE staining and histological examination. Bioluminescence images were collected to assess the growth and metastasis of implanted tumor cells. To quantify the in vivo bioluminescence signal, mice were anesthetized with isoflurane before in vivo imaging, and D-luciferin solution (in vivo imaging solutions, PerkinElmer, 150 mg/kg in PBS) was injected intravenously for systemic xenografts. Bioluminescence images were acquired with the IVIS Spectrum Imaging System (PerkinElmer) 2–5 min after injection, and the acquired images were quantified using the Living Image Software package (Perkin Elmer/ Caliper Life Sciences) by measuring the photon flux (photons/s/cm^2^/steradian) within a region of interest (ROI) drawn around the bioluminescence signal.

### Antagomir treatment

The antagomir and the miRNA negative control were synthesized by the Ribobio Company and implemented according to the manufacturer’s instructions (RiboBio, Guangzhou, China). A 10-nmol miR-10b-3p antagomir as well as the miRNA negative control in 0.1 ml saline buffer were locally injected into ESCC cell-forming tumor masses once every 5 days for 6 weeks. After the treatment, the ESCC cell-forming tumors were applied in the immunohistochemical assay. The tumor size was monitored by measuring the length (L) and width (W) with calipers every 5 days, and the volumes were calculated using the formula (L × W^2^)/2. The mice were killed by cervical dislocation on day 32, and the tumors were excised and snap-frozen for protein and RNA extraction.

### Evaluation of immunohistochemical staining

Tumor samples were fixed with 10% formalin in PBS, the paraffin-embedded 4 μm sections were baked at 65 °C for 60 min, and then rehydrated using graded alcohols. Each 4 μm tissue section was deparaffinized and rehydrated. The sections were deparaffinized and boiled in 10 mM citrate buffer (pH 6.0) for antigen retrieval, and incubated with fresh 3% H_2_O_2_ in methanol for 10 min at room temperature. Tissue sections were then incubated at 4 °C overnight with anti-FOXO3 (ab12162, Abcam, Cambridge, UK; 1:50 dilutions). Negative controls were prepared by replacing the primary antibodies with PBS. The tissues were washed three times in PBS for 5 min, and then incubated with secondary antibody for 30 min at 37 °C, and visualized with diaminobenzidine (Sigma). Two pathologists independently reviewed five random fields from each sample slide. FOXO3 expression was scored semi-quantitatively according to the percentage of positive cells and cytoplasmic/nuclear staining intensity. The results were assessed by two investigators. The percentage of positively stained cells was as follows: 0 (< 5% positive cells), 1 (6–25% positive cells), 2 (26–50% positive cells), 3 (51–75% positive cells), or 4 (> 75% positive cells). The cytoplasmic/nuclear staining intensity was categorized as follows: 0 score, negative; 1 score, buff; 2 score, yellow; and 3 score, brown. Optimal cut-off values for this assessment system were identified as follows: high expression of FOXO3 was defined as an expression index score of 5, while low expression as an expression index score of < 5. IHC staining images were captured at 100×, 200× and 400× under a microscope (Olympus).

### Statistical analysis

All values reported in the paper are expressed as the means ± SD, and all error bars represent the standard deviation of the mean. Student’s t-test, the χ2 test and repeated measures ANOVA were used to determine significance. The log-rank test was used to analyze the effect of clinical variables and miRNAs on patients’ OS. The Cox regression model was used to analyze the effect of the related factors on the survival time of patients with ESCC. Receiver operating characteristic (ROC) curves and the area under the ROC curve (AUC) were used to assess the feasibility of using serum miRNA as a diagnostic tool for detecting ESCC. CpG unit methylation data for miR-10b-3p from 18 pairs of ESCC tissues were used for stratified cluster analysis by Cluster 3.0 and Tree View software. A Wilcoxon test was also conducted to compare miR-10b-3p expression between ESCC and normal esophageal cancer tissues. *P* < 0.05 was considered statistically significant. Statistical analyses were performed using SPSS 16.0 software (SPSS Inc., USA).

## Results

### miR-10b-3p expression in human ESCC is increased and significantly correlated with poor survival

**Fig. 1:**
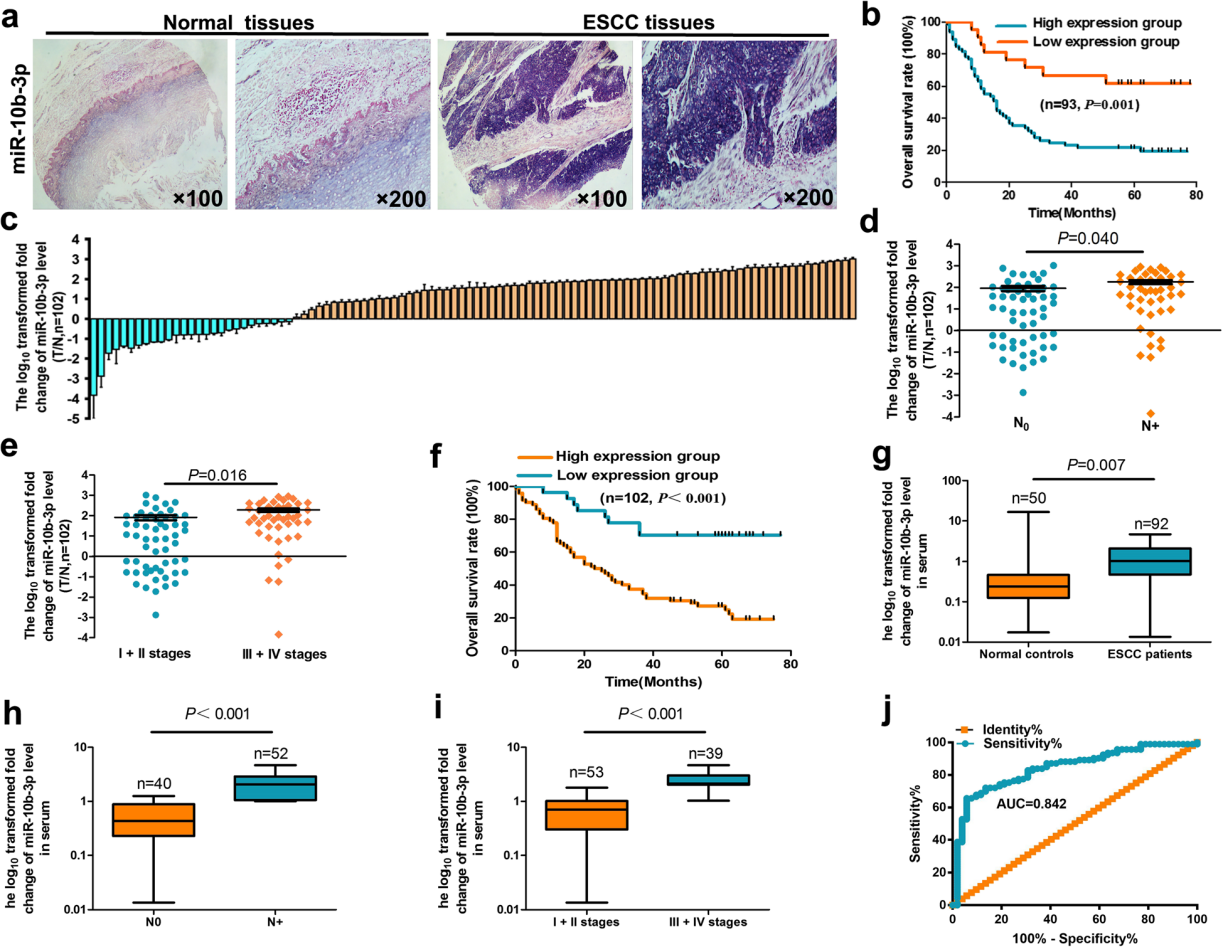
Relative miR-10b-3p expression levels in ESCC tissues and serum and their clinical significance. **a** Expression levels of miR-10b-3p in 93 paired ESCC and corresponding noncancerous tissues as measured by in situ hybridization. **b** Kaplan–Meier overall survival curves of high and low miR-10b-3p expression in 93 cases of ESCC. **c** Quantitation of miR-10b-3p was performed using qRT-PCR in 102 paired ESCC (T) and corresponding normal tissues (N). The fold changes were calculated by relative quantification (2^-∆Ct^, U6 as the internal control). **d**-**e**, miR-10b-3p expression was detected in lymph node metastasis (**d**) and different clinical stages (**e**) of ESCC. **f** Kaplan-Meier curves depicting overall survival according to the expression of miR-10b-3p as validation. **g** The expression level of serum miR-10b-3p in 92 ESCC patients and 50 healthy controls was measured by qRT-PCR and normalized to U6. h-i, miR-10b-3p expression was detected in lymph node metastasis (**h**) and different clinical stages (**i**). **j** Receiver operating characteristic (ROC) curve analysis of the miR-10b-3p assay ratio for detecting ESCC patients. Each experiment was performed in triplicate

To determine the potential functions of miR-10b-3p in ESCC pathogenesis, we analyzed miR-10b-3p expression in 93 pairs of ESCC tissues compared with that in normal esophageal tissues using an in situ hybridization method. miR-10b-3p expression was significantly upregulated in the tumor tissue samples compared with the controls (Fig. 1a, Table 1, *P* < 0.05). We further analyzed the relationship between clinicopathologic features and miR-10b-3p expression levels in ESCC cases. Importantly, we found that upregulation of miR-10b-3p expression was associated with lymph node metastasis and clinical stages (Table 1, *P* < 0.05). Clinically, the Kaplan–Meier test indicated that patients with miR-10b-3p overexpression exhibited significantly shorter survival times (Fig. 1b, *P* = 0.01). Age, gender, T stage, histological type, N stage, clinical stage and miRNA signature were used as covariates. Multivariate Cox regression analyses were used to investigate the independent prognostic value of the miR-10b-3p signature (Table 2, *P* < 0.01).

To validate whether miR-10b-3p expression is increased in ESCC, qRT-PCR was used to examine mature miR-10b-3p levels in human ESCC tissues and normal esophageal tissues. We found that miR-10b-3p levels in 102 ESCC tissues were markedly superior to those in normal esophageal tissues (Fig. 1c, Table 1, *P* < 0.05), particularly in cancer tissues with lymph node metastasis and advanced clinical stages of ESCC (Fig. 1d, e; Table 1, *P* < 0.05). Kaplan-Meier survival analysis also revealed that miR-10b-3p overexpression was associated with poor prognosis in patients with ESCC (Fig. 1f, *P* < 0.01). The miRNA signatures were observed to be independent prognostic factors related to overall survival (OS) by multivariate Cox regression analysis (Table 2, *P* < 0.01).

We used the qRT-PCR method to assess the expression level of serum miR-10b-3p. The expression of the miRNA was significantly higher in ESCC patients than in the normal controls (Fig. 1g, Table 3, *P* < 0.01). The results also revealed that serum miR-10b-3p was negatively associated with lymph node metastasis and advanced clinical stages of ESCC (Fig. 1h, i, Table 3, *P* < 0.01). We then produced ROC curves for ESCC diagnosis by serum miR-10b-3p levels and calculated the area under the curve as well as the sensitivity and specificity of all thresholds. The area under the curve for plasma miR-10b-3p was 0.842, indicating that there was a statistically significant difference in ESCC diagnosis by using serum miR-10b-3p as a marker (Fig. 1j).

### DNA hypomethylation results in miR-10b-3p overexpression in ESCC

**Fig. 2:**
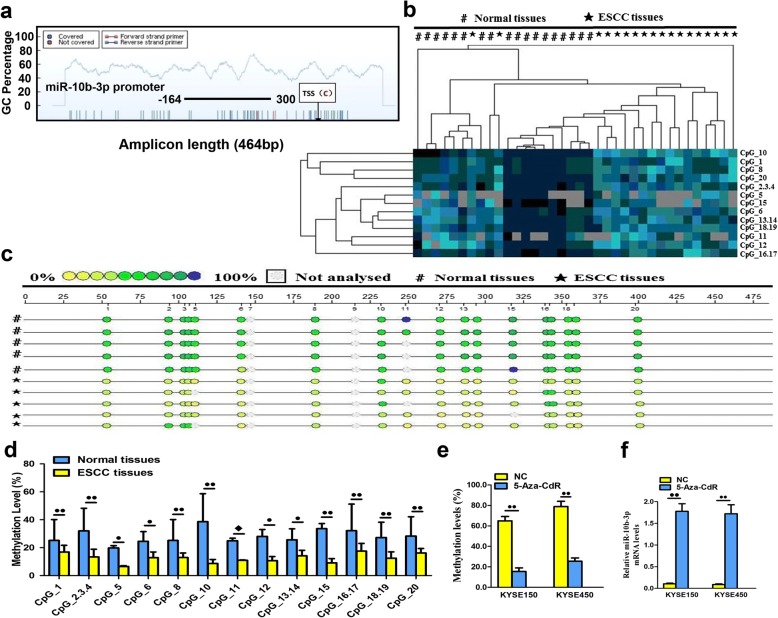
DNA methylation status of miR-10b-3p. **a** Genomic structure and distribution of miR-10b-3p CpG dinucleotides over the transcription start site (TSS). **b** The positions and orientation of the MassARRAY primers are indicated by horizontal black bars. Each column represents a sample. Each row displays the clustering of CpG units, which are a single CpG site or a combination of CpG sites. The color gradient between blue and yellow indicates methylation of each miR-10b-3p unit in each sample ranging from 0 to 100%. Gray represents technically inadequate or missing data. **c** Gene location, amplicon size, and place of CpG sites in the amplicon. Methylation profile of CpG sites for the miR-10b-3p gene. The color of the circles is related to the percentage of methylation at each CpG site. Boxes indicate the different methylation patterns between 18 ESCC samples and corresponding normal tissues. **d** Evaluation of CpG methylation within the miR-10b-3p promoter. The distribution of 13 analyzed CpG units within miR-10b-3p. **e** DNA methylation levels of the miR-10b-3p promoter region in 5-Aza-CdR-treated ESCC cells as detected by BSP assay. **f** Quantitation of the miR-10b-3p level after treatment with 5-Aza-CdR in KYSE150 and KYSE450 cell lines. Each experiment was performed in triplicate. Data are presented as the mean value ± SD. ^●^, *P* < 0.05; ^●●^, *P* < 0.01; ^◆^, *P* > 0.05

The MassARRAY System allows quantitative high-throughput detection and analysis of a single CpG site methylation within a target fragment. A single CpG site or a combination of CpG sites form a CpG unit. The miR-10b-3p promoter is located in a typical CpG site, suggesting the possible involvement of DNA methylation in the regulation of miR-10b-3p transcription (Fig. 2a). The amplicon detected in the promoter regions of miR-10b-3p was 464 base pairs in length and contained 19 CpG sites that can be divided into 13 CpG units. An obvious hierarchical clustering analysis was used to provide an equitable view of the relationships between ESCC and CpG units (Fig. 2b). The CpG methylation levels of the samples could be identified based on color for each miR-10b-3p CpG unit in each sample. The patterns observed in the cluster analysis indicated that the methylation status of miR-10b-3p in ESCC tissues was notably different from that in normal esophageal cancer tissues. We also found that the densities of methylated CpG dinucleotides were higher in normal tissues than in ESCC tissues (Fig. 2c). Lastly, we assessed the methylation level of each CpG unit within the miR-10b-3p promoter and found that 12 CpG units (except for CpG_11) were more highly methylated in normal esophageal tissues than in ESCC tissues (Fig. 2d, *P* < 0.05 or *P* < 0.01, respectively). A nonparametric test showed that apart from CpG_11, the mean methylation levels at CpG_1, CpG_2.3.4, CpG_5, CpG_6, CpG_8, CpG_10, CpG_11, CpG_12, CpG_13.14, CpG_15, CpG_16.17, CpG_18.19 and CpG_20 were all significantly higher in normal esophageal tissues (mean methylation = 40.17, 58.61, 23.11, 33.05, 33.50, 37.33, 51.22, 38.27, and 42.17%, respectively) than in ESCC (mean methylatio*n* = 21.72, 18.89, 7.17, 16.94, 16.56, 10.8, 11.83, 13.72, 18.17, 12.22, 23.06, 17.06, and 19.44%, respectively; *P* < 0.05). For the purpose of the result, 5-aza-2′-deoxycytidine (5-aza-CdR), the demethylation agent, was used to reverse methylation. The methylation level of miR-10b-3p was obviously inactivated in KYSE-150 (64.92%) and KYSE-450 (78.85%) cell lines compared with two corresponding untreated two cell lines (15.44 and 25.56%, respectively) with downregulation of methylation when treated with 5-aza-CdR (Fig. 2e*, P* < 0.01). Correspondingly, there were lower expression levels of miR-10b-3p in KYSE150 and KYSE450 cell lines treated with 5-aza-CdR compared to two untreated cell lines, which were negatively correlated with methylation status in ESCC cell lines (Fig. 2f*, P* < 0.01). There was direct evidence that the overexpression of miR-10b-3p in ESCC tissues was correlated with promoter hypomethylation, and demethylation of the promoter genes could upregulate the expression of miR-10b-3p.

### miR-10b-3p has positive effects on ESCC cell growth and metastasis

**Fig. 3:**
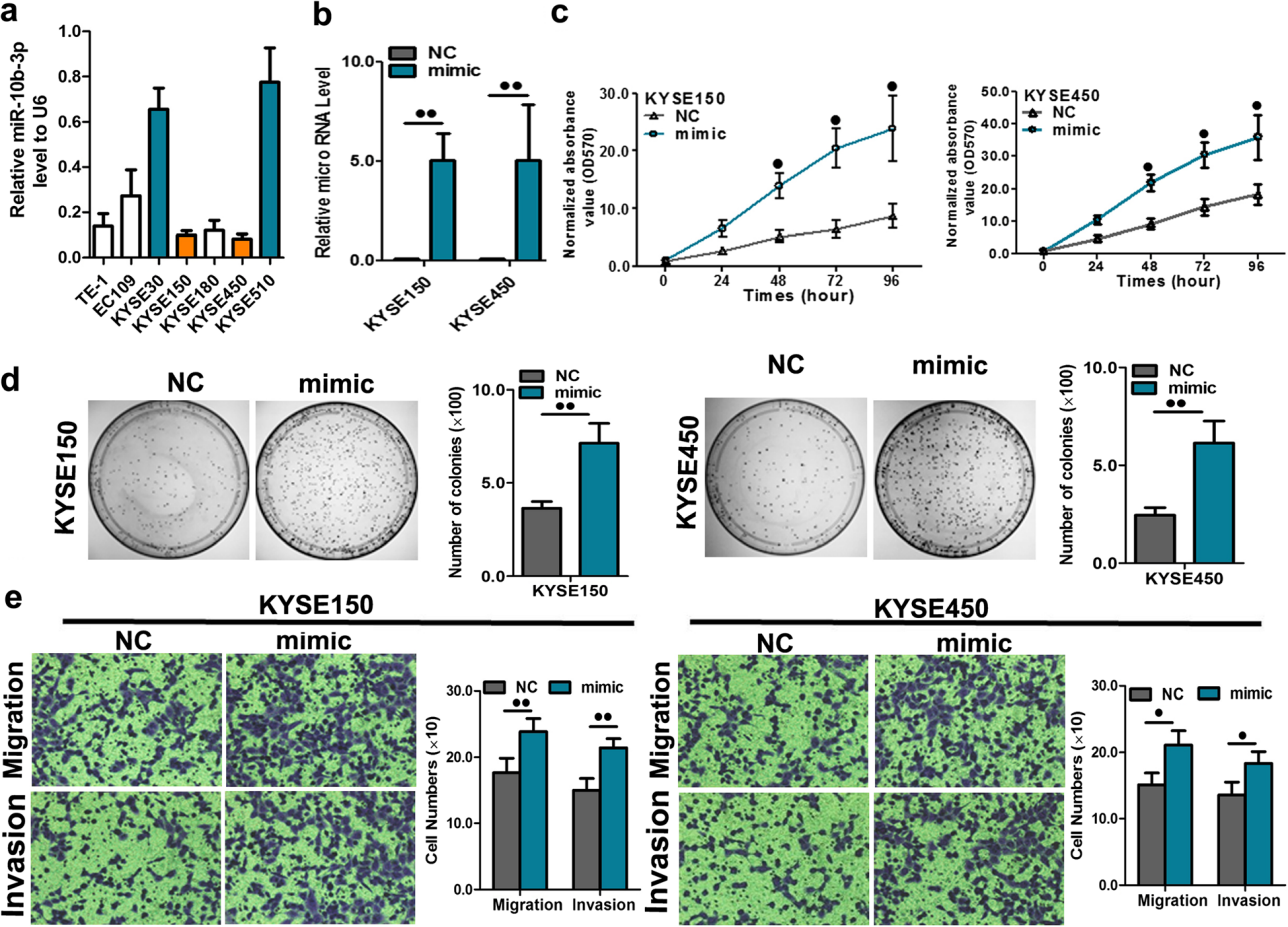
miR-10b-3p overexpression promoted cell proliferation, colony formation and migration. **a** RNA level of miR-10b-3p in 7 ESCC cell lines. **b** Quantitation of the miR-10b-3p level after transfection of the miR-10b-3p mimic in KYSE150 and KYSE450 cell lines. **c** The cell growth curve was measured by MTS after transfection of the miR-10b-3p mimic in KYSE150 and KYSE450 cell lines, and the OD 570 was normalized to the star point (0 h). **d** Representative images and quantitation of colony formation were performed after transfection of the miR-10b-3p mimic in KYSE150 and KYSE450 cell lines. **e** Representative images and quantitation of the transwell assay were performed after transfection of the miR-10b-3p mimic in KYSE150 and KYSE450 cell lines. Each experiment was performed in triplicate. Data are presented as the mean value ± SD. ^●^, *P* < 0.05; ^●●^, *P* < 0.01

Owing to the lower expression of miR-10b-3p in the KYSE150 and KYSE450 cell lines among seven ESCC cell lines (Fig. 3a), these two cell lines were selected for a forced overexpression study. To further investigate the role of miR-10b-3p in the regulation of ESCC cell proliferation, colony formation, invasion and migration, KYSE150 and KYSE450 cells were transfected with a miR-10b-3p mimic, and miR-10b-3p levels were then examined using qRT-PCR. The efficiency of transfection was verified by a significant increase in miR-10b-3p expression in KYSE150 and KYSE450 cells as determined by qRT-PCR (Fig. 3b, *P* < 0.01). We found that high exogenous expression of miR-10b-3p remarkably promoted proliferation, colony formation, migration, and invasion of KYSE150 and KYSE450 cells (Fig. 3c, d, e; *P* < 0.05 or *P* < 0.01). However, upon miR-10b-3p overexpression, the percentages of KYSE150 and KYSE450 cells in the early and late phases of apoptosis clearly decreased compared with the percentages measured in the controls (Additional file 1: Figure S1).

**Fig. 4:**
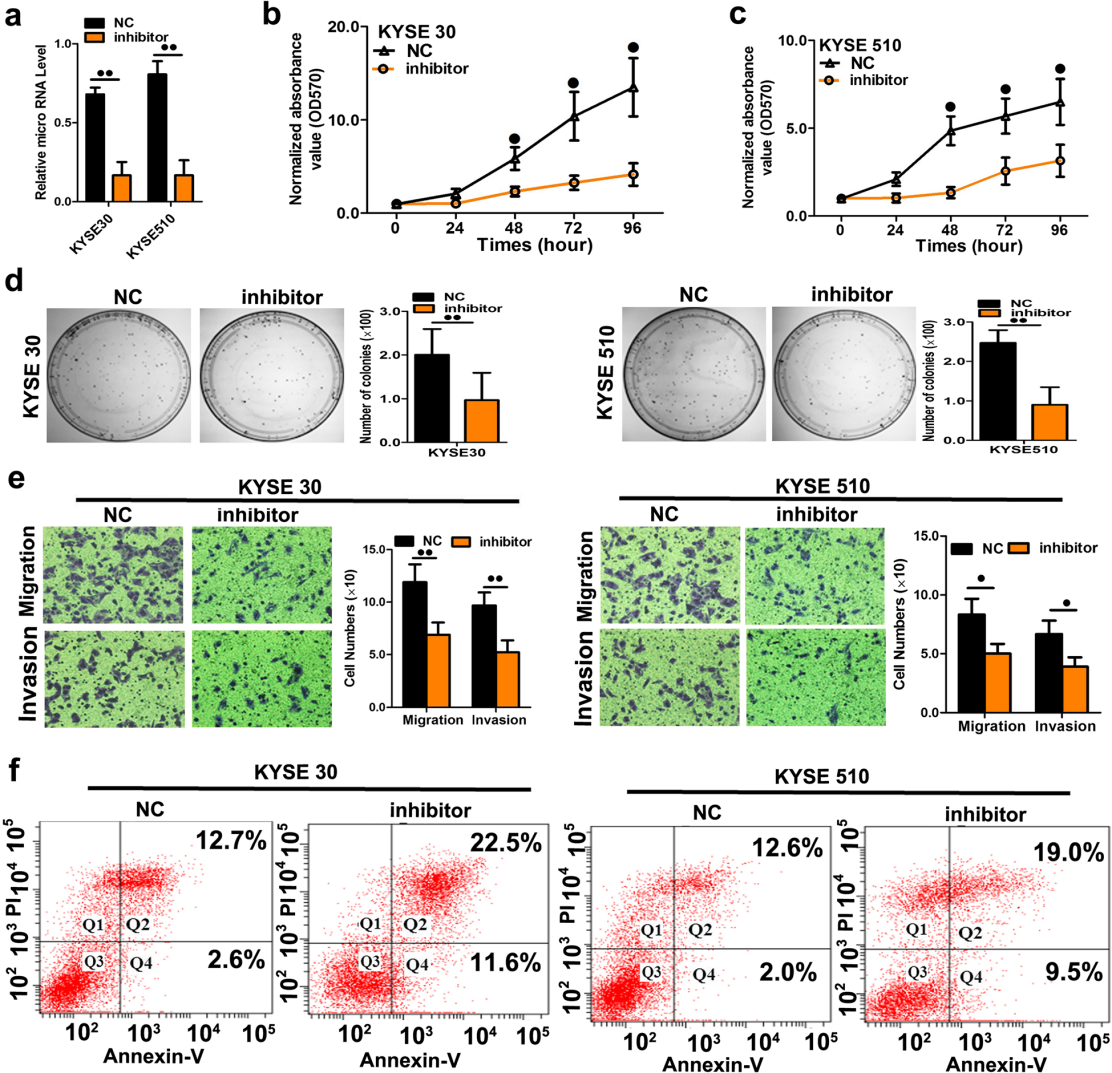
Repression of miR-10b-3p expression significantly inhibited cell growth, colony formation, and migration in ESCC cells. **a** Quantitation of the miR-10b-3p level after transfection of the miR-10b-3p inhibitor in KYSE30 and KYSE510 cell lines. **b**-**c** The cell growth curve was measured by MTS after transfection of the miR-10b-3p inhibitor in KYSE30 and KYSE510 cell lines, and the OD 570 was normalized to the star point (0 h). **d** Representative images and quantitation of colony formation were performed after transfection of the miR-10b-3p inhibitor in KYSE30 and KYSE510 cell lines. **e** Representative images and quantitation of the transwell assay were performed after transfection of the miR-10b-3p inhibitor in the KYSE30 and KYSE510 cell lines. **f** miR-10b-3p inhibitor induced apoptosis. Each experiment was performed in triplicate. Data are presented as the mean value ± SD. ^●^, *P* < 0.05; ^●●^, *P* < 0.01

Next, we transfected ESCC cells with inhibitors of miR-10b-3p to confirm the opposite effects of miR-10b-3p mimic transfection (Fig. 4a, *P* < 0.01). As expected, downregulation of miR-10b-3p using these inhibitors decreased the malignant phenotype of KYSE30 and KYSE510 cells in vitro, including cell growth (Fig. 4b, c; *P* < 0.05), colony formation (Fig. 4d, *P* < 0.01), cell migration and cell invasion (Fig. 4e, *P* < 0.05 or *P* < 0.01). To explore the possible mechanism underlying the phenotype of cell growth caused by overexpression of miR-10b-3p, apoptosis analysis was performed. Upon downregulation of miR-10b-3p, the percentages of KYSE30 and KYSE510 cells in the early and late phases of apoptosis clearly increased compared with the percentages measured in the controls (Fig.4f), indicating that miR-10b-3p downregulation resulted in decreased apoptosis in ESCC cells.

### miR-10b-3p targets FOXO3 to contribute to proliferation and metastasis

**Fig. 5:**
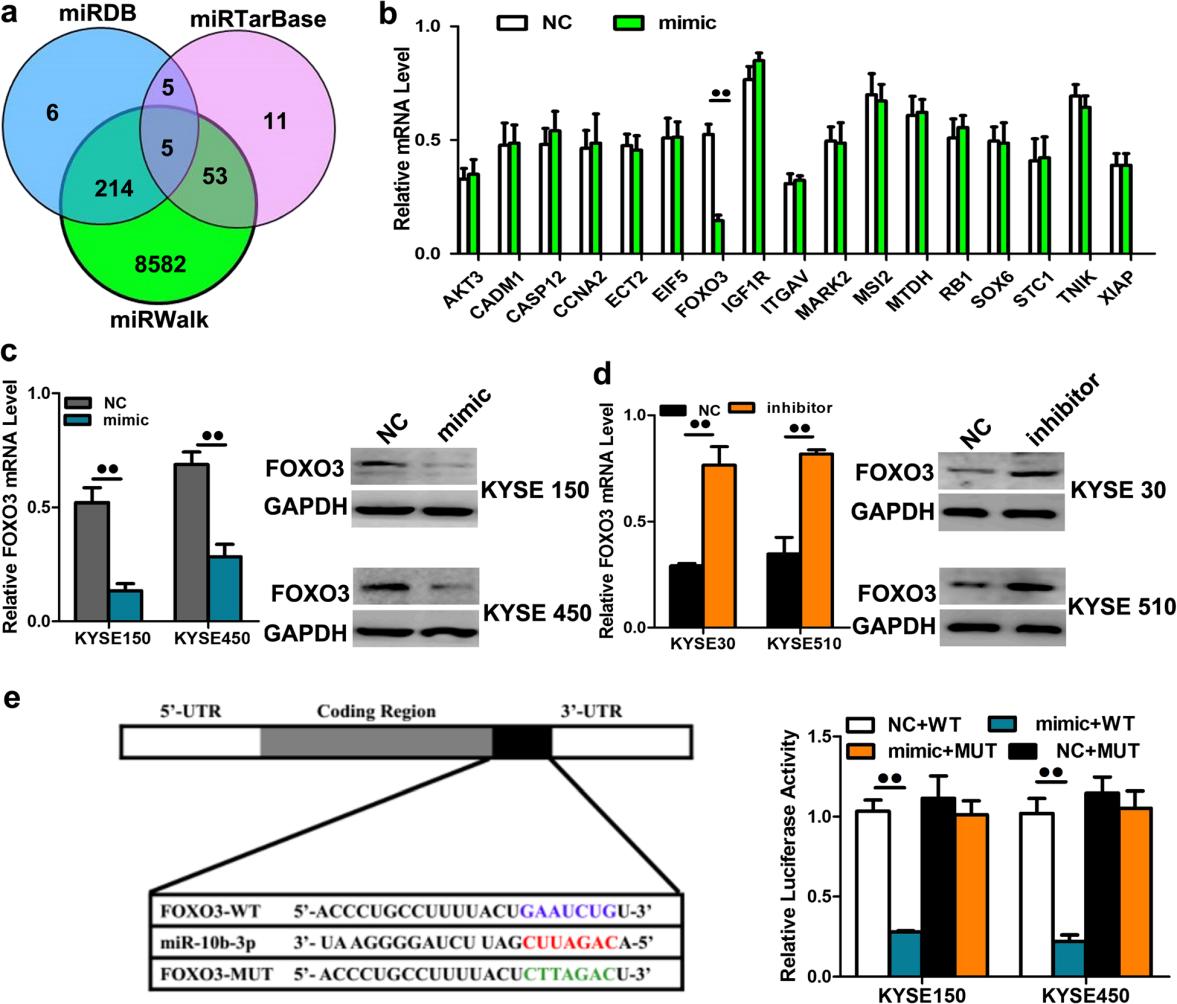
FOXO3 was one direct target gene of miR-10b-3p. **a**-**b** FOXO3 was identified as potential regulatory targets of miR-10b-3p by considering the downregulation of genes using prediction tools and qRT-PCR. **c** The expression levels of FOXO3 mRNA and protein were measured by qRT-PCR and western blot analysis using GAPDH as the loading control after transfection of the miR-10b-3p mimic in the KYSE150 and KYSE450 cell lines, respectively. **d** The expression levels of FOXO3 mRNA and protein were measured by qRT-PCR and western blot analysis using GAPDH as the loading control after transfection of miR-10b-3p inhibitors in the KYSE30 and KYSE510 cell lines, respectively. **e** Dual-luciferase reporter assay. The relative luciferase activity was normalized to the Renilla luciferase activity assay after cotransfection with the miR-10b-3p mimic and miR-RB-REPORT constructs containing WT or MUT FOXO3 3’UTR region in KYSE150 and KYSE450 cell lines. Each experiment was performed in triplicate. Data are presented as the mean value ± SD. ^●^, *P* < 0.05; ^●●^, *P* < 0.01

To explore the mechanism by which miR-10b-3p regulates ESCC cell progression, we searched for potential downstream regulatory targets of miR-10b-3p using several bioinformatics methods, including miRDB, miRTarBase and miRWalk (Fig. 5a). Then, several candidate genes involved in cell proliferation, apoptosis and invasion-metastasis were annotated by Gene Ontology (GO) terms and verified by qRT-PCR. We found that the 3’UTR of FOXO3 mRNA contains sequences that are potential targets of miR-10b-3p (Fig. 5b, *P* < 0.01) (Additional file 2: Table S1). To verify whether FOXO3 is a direct target of miR-10b-3p, KYSE150 and KYSE450 cells transfected with miR-10b-3p mimic presented remarkably downregulated mRNA and protein levels of FOXO3 (Fig. 5c, *P* < 0.01). We also transfected KYSE30 and KYSE510 cells with inhibitors of miR-10b-3p to confirm the results of mimic transfection. As expected, downregulation of miR-10b-3p using inhibitors could enhance the FOXO3 mRNA and protein levels in KYSE30 and KYSE510 cells (Fig. 5d, *P* < 0.01). We next applied the dual-luciferase reporter assay to reveal the regulation of miR-10b-3p by FOXO3. The fragments containing the miR-10b-3p binding sequence or mutated sequence in the 3’UTR region of FOXO3 were cloned into the pmiR-RB-REPORT vector luciferase reporter. These reporter constructs were cotransfected with miR-10b-3p mimic or miR-NC into KYSE150 and KYSE450 cells, and the luciferase activities were subsequently measured. The miR-10b-3p mimic significantly suppressed the luciferase activity of pmiR-RB-REPORT-FOXO3–3’UTR (Fig. 5e, *P* < 0.01), while miR-NC had no inhibitory effect on pmiR-RB-REPORT-FOXO3–3’UTR. The miR-10b-3p inhibition of pmiR-RB-REPORT-FOXO3–3’UTR was sequence specific because the luciferase activities of pmiR-RB-REPORT-FOXO3-mut did not decrease in the presence of miR-10b-3p. Taken together, these results suggest that miR-10b-3p can directly target the 3’UTR of miR-10b-3p.

**Fig.6:**
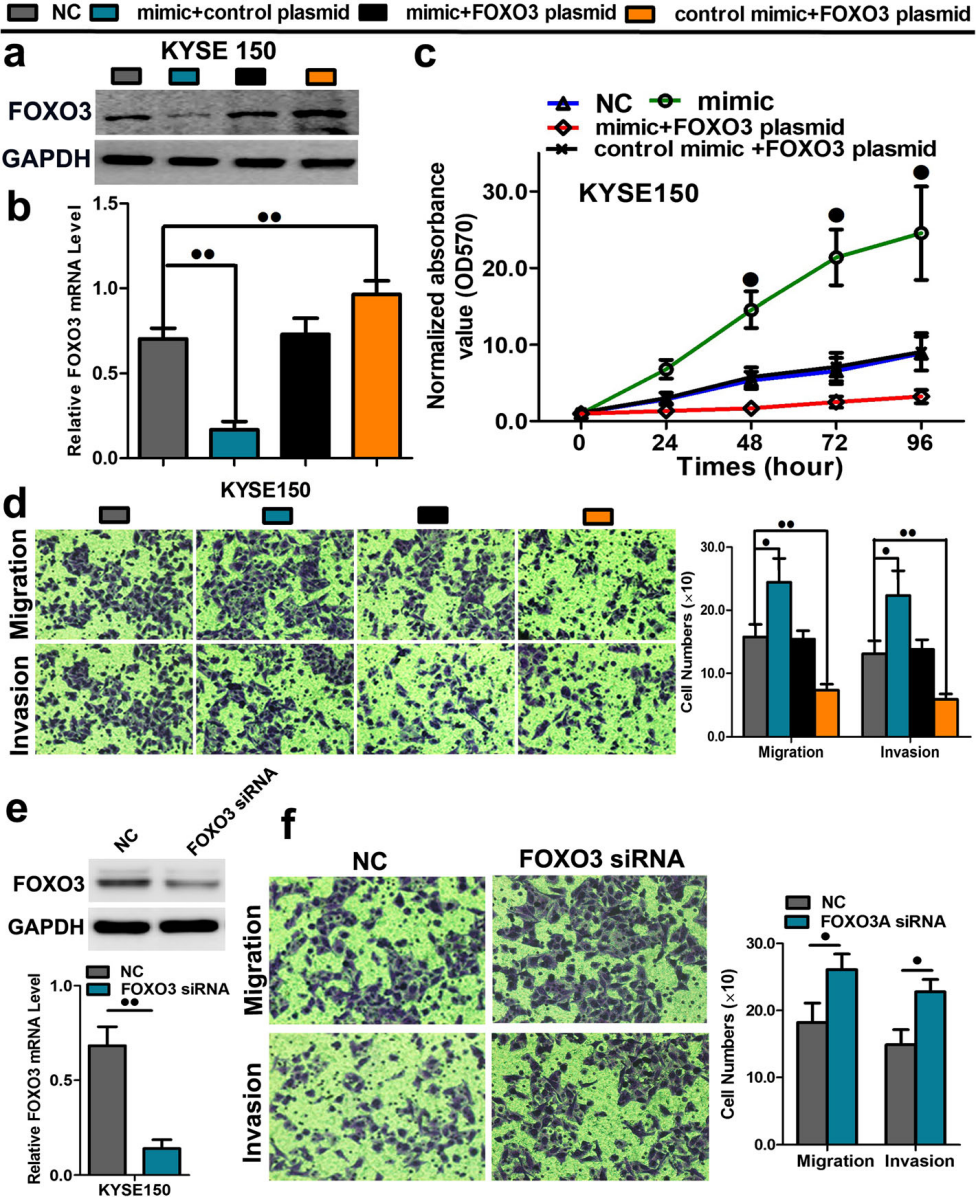
A rescue assay was further performed to confirm that FOXO3 was the functional target of miR-10b-3p. **a**-**b** The mRNA and protein levels of FOXO3 in KYSE150 and KYSE450 cell lines cotransfected with miR-10b-3p mimic and pEGFP-C1 plasmid containing FOXO3 CDS sequence. **c** The cell growth curve was measured by MTS cotransfected with miR-10b-3p mimic and FOXO3 plasmids in KYSE 150 cell lines, and the OD 570 was normalized to the star point (0 h). **d** Transwell assay of cells cotransfected with miR-10b-3p mimic and FOXO3 plasmids. **e** The expression of FOXO3 at the mRNA and protein level post siRNA silencing in KYSE150 cells. **f** Representative images and quantification of transwell assay after the transfection of FOXO3 siRNA into the KYSE150 cell lines. Each experiment was performed in triplicate. Data are presented as the mean value ± SD. ^●^, *P* < 0.05; ^●●^, *P* < 0.01

A rescue experiment was performed to confirm that FOXO3 was the functional target of miR-10b-3p in KYSE150 cells. The evidence was obtained from the observation that FOXO3 mRNA and protein (endogenous) expression in ESCC cells was abolished by mimic transfection and recovered by transfection of both pEGFP-N1-FOXO3 expression constructs (Fig. 6a, b; *P* < 0.01). The results showed that cell proliferation, migration and invasion created by mimic transfection were reversed by transfection of both expression constructs (Fig. 6c, d; Additional file 3: Figure S2; *P* < 0.05 or *P* < 0.01). Moreover, The results showed that migration and invasion created by mimic transfection were reversed by transfection of both expression constructs (Fig. 6d and e). The cell growth curve was measured by MTS showed decreased cell proliferation rates after transfection of the FOXO3 plasmid overexpression in KYSE30 and KYSE510 cell lines (Additional file 4: Figure S3a, b; *P* < 0.05). The apoptosis was measured by FACS analysis also indicated increases of early and late phase of apoptosis in cells with FOXO3 plasmid overexpression in KYSE 30 and KYSE 510 cell lines (Additional file 4: Figure S3c, d). Furthermore, the expression of FOXO3 was silenced by siRNA transfection in KYSE150 cells, indicating that its expression was significantly attenuated at the mRNA and protein levels (Fig. 6d, *P* < 0.01). After FOXO3 silencing, migration and invasion abilities of ESCC cells were significantly increased (Fig. 6e, *P* < 0.01). These results further prove that FOXO3 is a downstream target of miR-10b-3p.

### miR-10b-3p accelerates tumor growth and metastasis in vivo

**Fig. 7:**
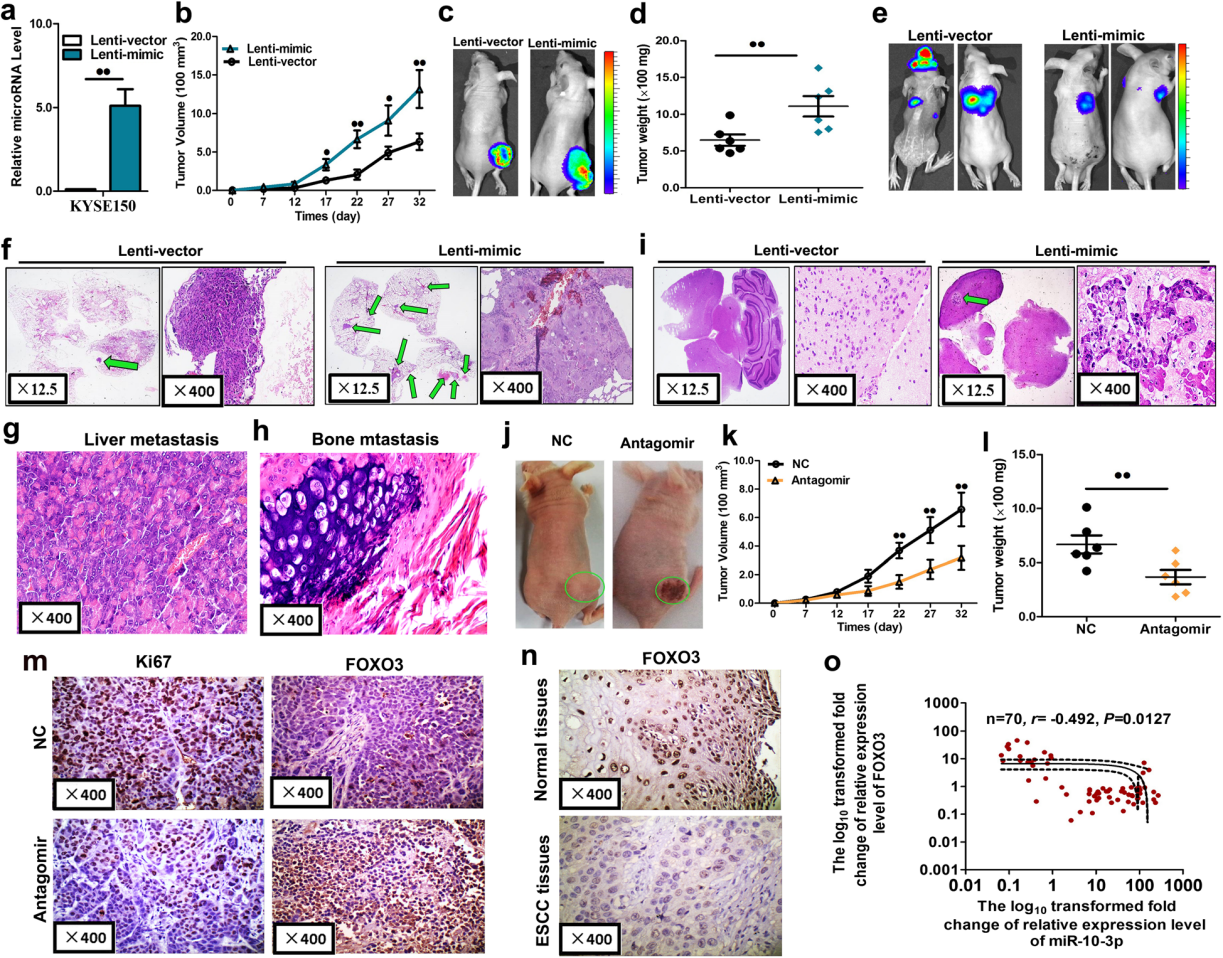
miR-10b-3p promoted tumor growth and metastasis in vivo. **a** Levels of miR-10b-3p in stable overexpressing KYSE150 cells (Lenti-mimic) and control cells (Lenti-vector). **b**-**d** Stable miR-10b-3p overexpressing KYSE150 cells were subcutaneously injected into nude mice to form solid tumors, and representative images of tumor volumes and weights were analyzed by in vivo luciferase imaging on the last day of analysis (*n* = 5 for each group). **e**-**i** The metastatic nodules were observed in the lungs, brains, liver, and bone of mice treated with stable miR-10b-3p overexpressing KYSE150 cells or control vector cells by the vein injection method. **j**-**l**, KYSE150 cells were subcutaneously injected into nude mice to form solid tumors and synchronously treated with miR-10b-3p antagomir or miR antagomir NC (*n* = 5 for each group); **a** 10-nmol miR-10b-3p antagomir as well as the miRNA negative control in 0.1 ml saline buffer was locally injected into nude mice to treat tumor mass once every 5 days for 5 weeks; tumor weight and volume in nude mice. **m** Immunohistochemical staining of Ki67 and FOXO3 in tumor tissues dissected from nude mice treated with miR-10b-3p antagomir miR or antagomir NC. **n** FOXO3 protein expression as measured by immunohistochemical staining in 103 ESCC samples and pair-matched esophageal tissues. **o** Spearman correlation analysis of the negative correlation between the expression of miR-10b-3p and FOXO3. Each experiment was performed in triplicate. Data are presented as the mean value ± SD. ^●^, *P* < 0.05; ^●●^, *P* < 0.01

Finally, we evaluated the effects of miR-10b-3p on the growth and metastasis of ESCC in nude mice. KYSE150 cells were transfected with either a lentiviral expression vector of miR-10b-3p (Lenti-mimic) or a negative control lentiviral vector (Lenti-vector). Efficient overexpression of miR-10b-3p in KYSE150 cells following lentiviral infection was verified by qRT-PCR (Fig. 7a, *P* < 0.01). Then, we injected these KYSE150 cells subcutaneously to generate transplanted tumors in BALB/c nude mice. Beginning on day 7 after implantation, the tumor lengths and widths were measured every 5 days to obtain 6 measurements. The tumor growth curve revealed significant acceleration in the miR-10b-3p-overexpressing group compared with that in the control group (Fig. 7b, *P* < 0.05 or *P* < 0.01). Subsequently, the tumors were dissected, and the exact sizes and weights were evaluated. The mean volume and mass of the tumors in the miR-10b-3p overexpressing group were significantly larger and heavier than those of the tumors in the control group (Fig. 7c, d; *P* < 0.01).

In addition, 10^6^ luciferase-labeled cells were injected intravenously by tail vein injection into the mice. After 6 weeks luciferase activity was used to evaluate tumor burden in all organs of nude mice. The lung, liver and bone metastasis burden was significantly higher in the mice injected with miR-10b-3p overexpressed cells compared with the control group (Fig. 7f, i, g, h). Especially, miR-10b-3p overexpression significantly increased the brain metastasis sites of ESCC cells through tail vein injection (Fig. 7i). All these results obtained from the mouse models suggest that miR-10b-3p plays important roles in ESCC growth and metastasis, particularly in brain metastasis.

To determine whether miR-10b-3p antagomir could inhibit the growth of ESCC in nude mice, we established a nude mouse tumorigenic model using KYSE150 cells. After 7 days, miR-10b-3p antagomir or miR antagomir NC was directly injected into the implanted tumor every 5 days. The tumor volume was measured every 5 days until day 32. The tumor volume and weight of mice treated with miR-10b-3p antagomir were significantly lower than those of mice treated with miR antagomir NC (Fig. 7h, i, j; *P* < 0.01). These results indicated that miR-10b-3p has therapeutic effects on ESCC cells in the nude mouse tumorigenic model.

The proliferative activities of the tumor cells were assessed by immunohistochemical staining for Ki-67 in FFPE tissues of xenograft tumors. The Ki-67 staining intensities were decreased in tumors from the miR-10b-3p antagomir group (Fig. 7k). A distinct increase in FOXO3 expression was observed in xenograft tumors treated with miR-10b-3p antagomir group compared with that in xenograft tumors treated with miR antagomir NC group (Fig. 7k). In the analysis of 103 paired tumor and adjacent nontumor tissue samples, we found that FOXO3 expression was significantly lower in tumor tissues than in adjacent nontumor tissues (Table 4, Fig. 7l). We determined the association of miR-10b-3p expression and FOXO3 expression levels in 103 ESCC tissue samples with Spearman correlation coefficient analysis. miR-10b-3p expression levels were inversely correlated with the expression levels of FOXO3 in the 103 ESCC specimens (Fig. 7m, *P* < 0.05).

**Fig. 8:**
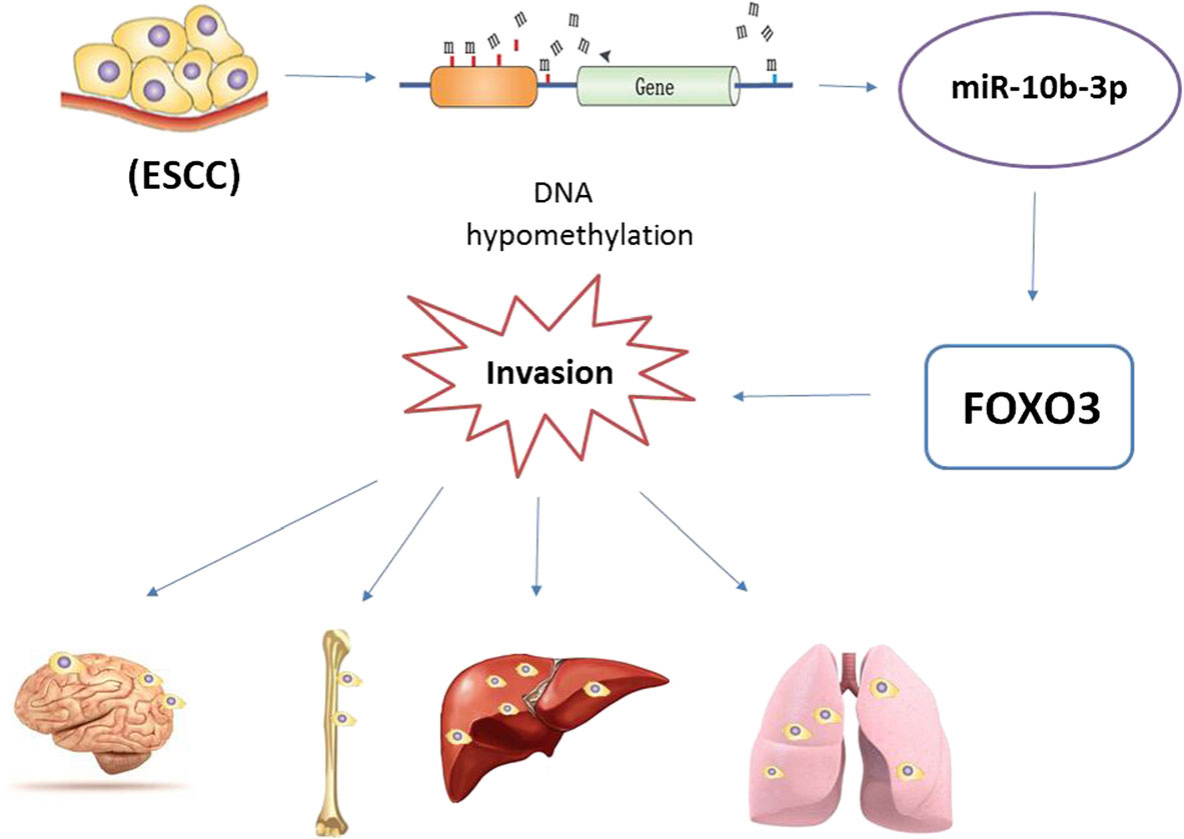
Schematic diagram summarizing how miR-10b-3p promotes tumor initiation and progression of esophageal cancer by regulating FOXO3

Collectively, our findings suggested upregulated expression of miR-10b-3p caused by promoter hypomethylation contributed to the progression of ESCC and miR-10b-3p suppresses tumor initiation and progression of esophageal cancer by regulating FOXO3 (Fig. 8).

## Discussion

The importance of miRNA functions and dysfunctions in various human cancers suggests that modulation of miRNA expression may serve as a novel diagnostic and therapeutic modality for cancers. Variations of the expression levels of microRNAs have been reported in ESCC tissues compared to their normal counterparts previously. The reduced expression of microRNAs such as miR-138 and miR-145 has been noted in ESCC tissues [18, 19]. And miR-21, miR-200c and miR-133a have been reported to be upregulated in esophageal cancer [19, 20]. In the present study, we explored the role of miR-10b-3p in ESCC progression. We identified miR-10b-3p as a novel oncogenic miRNA in ESCC. We found miR-10b-3p was significantly upregulated in esophageal cancer tissue samples and exhibited expression levels that were positively correlated with the clinical tumor stage and lymph node metastasis by both in situ hybridization and qPCR assays. We further explored the role of miR-10b-3p in ESCC progression and the underlying molecular mechanisms were elucidated.

Endogenous circulating miRNAs have attracted significant attention in the field for their potential uses in the diagnosis, prognosis and metastasis of cancers. Tumor-derived miRNAs were first described in plasma by Mitchell et al. [21]. Importantly, studies investigating plasma miRNAs comprise an extremely promising field for clinical application. The serum miR-10b-3p level has been demonstrated to be useful in delineating ESCC stages due to the increasing expression of miR-10b-3p in higher-stage cancers. Moreover, lymph nodes that contain metastatic tumors show upregulation of miR-10b-3p expression. An increasing body of evidences has suggested the presence of circulating miRNAs and their potential use as novel biomarkers for cancers, such as breast cancer [22], lung cancer [23], pancreatic cancer [24, 25], gastric cancer [26, 27], and colorectal cancer [28]. There have been three reports on the role of circulating miRNAs in plasma/serum patients with ESCC [29–31]. Additionally, our results showed that the high expression of miR-10b-3p might be closely associated with poor overall survival. Zhou et al. also demonstrated that a high expression level of miR-103/107 in serum was an independent poor prognostic factor in patients with ESCC, as determined by multivariate Cox regression [32]. The serum-based approach is more advantageous than tumor tissue biopsy, which uses an invasive procedure as the main tool for ESCC risk assessment. Our study is the first to report the diagnostic and prognostic value of tissue and serum miR-10b-3p levels.

DNA hypomethylation-mediated activation of oncogenic miRNAs is an important mechanism of tumorigenesis by the silencing the tumor suppressive genes. This phenomenon has been reported for several miRNAs, such as miR-196b, miR-9-1, let-7a-3, miR-106a, and miR-146a [33–36]. To identify whether miR-10b-3p methylation occurs in ESCC tissues, we measured the methylation levels of all our samples and found that the methylation status of ESCC tissues was notably lower than that of normal esophageal tissues. We further verified that demethylation by 5-aza-CdR assay which dramatically augmented the expression of miR-10b-3p in ESCC cell lines. This is the first to reveal DNA methylation in regulating miR-10b-3p expression.

To better understand the role of miR-10b-3p in regulation of ESCC progression, we investigated its biological functions with both in vitro cellular assays and in vivo mouse experiments. Ectopic overexpression of miR-10b-3p promoted the proliferation, colony formation, and invasion of ESCC cells, whereas down-regulation of miR-10b-3p had the opposite effects. Consistently, miR-10b-3p overexpression induced more robust tumor formation and lung metastasis in ESCC mice xenograft model. These results indicated that miR-10b-3p plays a crucial role in the growth and metastasis of ESCC.

Accumulating evidences suggest miRNAs participate in tumor growth and metastatic processes, and a growing number of miRNAs have been found to be involved in ESCC progression [37]. Overexpression of miR-10b-3p in KYSE140 increased cell motility and invasiveness, and a significant upregulation of miR-10b in 40 human ESCC samples compared to their adjacent tissues has been reported [12]. Li et al. reported that miR-377 plays an important role in suppressing tumor initiation and progression of ESCC, which may represent a promising noninvasive diagnostic and prognostic biomarker for patients with ESCC [38]. miR-638 acted as an oncogene and could cause a significant increase in tumor cell metastasis in vitro and esophageal metastasis in a nude mouse xenograft model [39]. Similarly, positive roles of miR-10b-3p in tumor growth and metastasis have been demonstrated in previous studies [40]. Collectively, these results support our findings that miR-10b-3p functions as an oncogene in ESCC, and this miRNA may be a promising target to tackle this cancer.

The mechanisms by which miRNAs alter gene expression remain controversial, but most studies have suggested that miRNAs are primarily processed by the RNA-mediated interference machinery to trigger partial or complete mRNA degradation of target genes. Our bioinformatics analysis revealed that miR-10b-3p could bind to the 3′UTR of FOXO3, and we observed that the expression of FOXO3 could be repressed by ectopic miR-10b-3p overexpression. In addition, miR-10b-3p-induced increased cell proliferation could be at least partially accounted to the reduced apoptosis caused by FOXO3 repression.

FOXO3 is a member of the forkhead box transcription factor O subfamily. FOXO3, together with other members, such as FOXO1 and FOXO4, participate in cell functions related to cell cycle arrest, induction of apoptosis, and oxidative and cellular stress [41]. In general, FOXO3 is known to inhibit cell cycle progression and to promote cell death, and it has been studied as an important inhibitor of cancer cell progression [42]. Previous studies have also reported that FOXO3 can antagonize the functions of FOXM1, which contributes to cancer initiation, progression, and drug resistance [43, 44]. In this study, we found that overexpression of miR-10b-3p inhibited FOXO3 protein expression by directly binding to its 3’UTR, which resulted in increased proliferation and metastasis in ESCC cells. In addition, we verified FOXO3 is a downstream target of miR-10b-3p by showing an inversed correlation of the expression of miR-10b-3p and FOXO3 in human ESCC samples. The ability of FOXO3 overexpression to counteract the pro-invasion effects of miR-10b-3p clearly indicates the importance of their relationship in ESCC metastasis. Our IHC analysis also indicated FOXO3 levels were lower in esophageal cancer tissues than in normal esophageal tissues. And low FOXO3 levels correlated with the increased lymph node metastasis ratios and advanced clinical stages. These combinatory functional studies added additional surpports to our prediction that FOXO3 is an important downstream target of miR-10b-3p and plays important roles in the progression and metastasis of ESCC. However, limitations still exist in this study. Future studies are required to further clarify whether other factors participated in miR-10b-3p signal axis and affected cancer progression. For example, Some research indicated that up-regulation of miR-10b-3p could promote the progression of HCC cells by suppressing CMTM5 expression.

## Conclusions

In conclusion, we observed upregulation of miR-10b-3p in esophageal tissues and demonstrated that miR-10b-3p may act as an independent predictor of OS for ESCC patients; we also discovered the hypomethylation of the promoter of miR-10b-3p is the primary cause for its overexpression. In addition, we found the serum miR-10b-3p level may serve as a novel and stable biomarker for patients with ESCC. We further found FOXO3 is the downstream target of miR-10b-3p which has a potent effect that promotes ESCC growth and metastasis. Collectively, our findings suggest that miR-10b-3p functions as an oncogene in ESCC that can be further explored as a biomarker and potential therapeutic target for ESCC [45].

## Additional files

Additional file 1: **Figrue S1.** miR-10b-3p overexpression significantly inhibited cell apoptosis in ESCC cells. a miR-10b-3p reduced apoptosis in KYSE 150 cells. a miR-10b-3p reduced apoptosis in KYSE 450 cells. Each experiment was performed in triplicate. (JPG 890 kb)

Additional file 2: **Table S1.** The Specific primers of potential targets. (DOC 38 kb)

Additional file 3: **Figure S2.** A rescue assay was further performed to confirm that FOXO3 was the functional target of miR-10b-3p in KYSE450 cells. a The cell growth curve was measured by MTS cotransfected with miR-10b-3p mimic and FOXO3 plasmids in KYSE 450 cell lines, and the OD 570 was normalized to the star point (0 h). b Transwell assay of KYSE 450 cells with cotransfected with miR-10b-3p mimic and FOXO3 plasmids. (JPG 5228 kb)

Additional file 4: **Figure S3.** FOXO3 plasmid overexpression significantly inhibited cell proliferation and promoted apoptosis in ESCC cells. a-b The cell growth curve was measured by MTS after transfection of the FOXO3 plasmid overexpression in KYSE30 and KYSE510 cell lines, and the OD 570 was normalized to the star point (0 h). c-d The cell apoptosis was measured by FACS analysis FOXO3 plasmid overexpression in KYSE 30 and KYSE 510 cell lines. Each experiment was performed in triplicate. (JPG 1648 kb)
