# Correction to: Promoter hypomethylation mediated upregulation of MicroRNA-10b-3p targets FOXO3 to promote the progression of esophageal squamous cell carcinoma (ESCC)

**DOI:** 10.1186/s13046-020-1525-0

**Published:** 2020-01-21

**Authors:** Yi-fang Lu, Jia-rui Yu, Zhao Yang, Guan-xia Zhu, Peng Gao, Huan Wang, Si-yuan Chen, Jie Zhang, Mei-yue Liu, Yi Niu, Xiao-mei Wei, Wei Wang, Feng-jin Ye, Li-xin Zhang, Yue Zhao, Guo-gui Sun

**Affiliations:** 1 Department of medicine, Tangshan gongren Hospital, Tangshan, China; 2 Department of Radiation Oncology, North China University of Science and Technology Affiliated People’s Hospital, Shengli Road, Tangshan, 063000 China; 3 Zhejiang Cancer Research Institute, Zhejiang Cancer Hospital, Hangzhou, 310022 China; 4 Wenzhou Medical College, Wenzhou, China; 5 Department of pathology, North China University of Science and Technology Affiliated People’s Hospital, Tangshan, China

**Correction to: J Exp Clin Cancer Res**

**https://doi.org/10.1186/s13046-018-0966-1**

In the original publication of this manuscript [1], Fig. 6 contains a repeated image in error (the left image of ‘Migration’ and the left image of ‘Invasion’). The misused pictures do not influence the data statistics; all of the original results remain unchanged. The revised Fig. 6 is shown below:

**Fig. 6:**
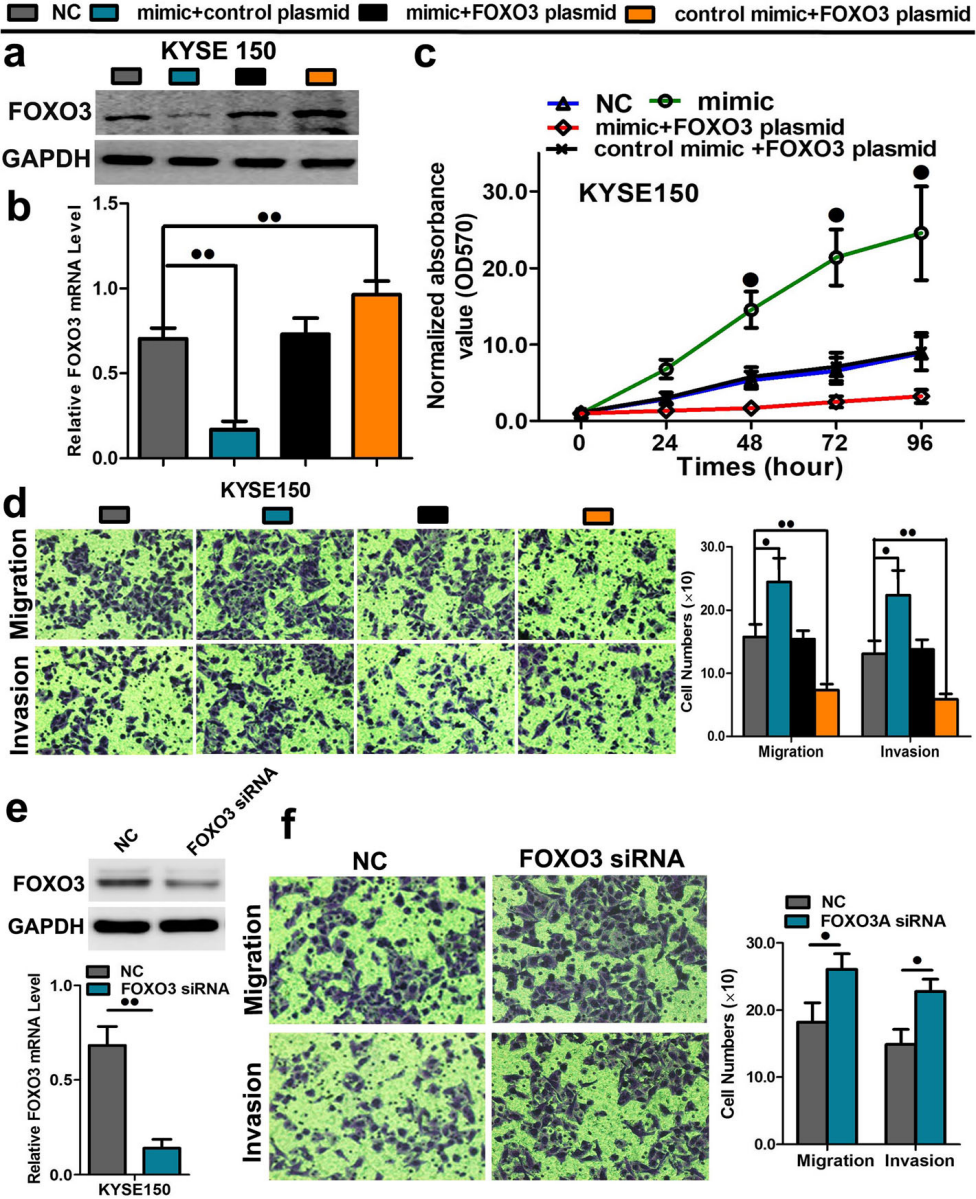
A rescue assay was further performed to confirm that FOXO3 was the functional target of miR-10b-3p. **a**-**b** The mRNA and protein levels of FOXO3 in KYSE150 and KYSE450 cell lines cotransfected with miR-10b-3p mimic and pEGFP-C1 plasmid containing FOXO3 CDS sequence. **c** The cell growth curve was measured by MTS cotransfected with miR-10b-3p mimic and FOXO3 plasmids in KYSE 150 cell lines, and the OD 570 was normalized to the star point (0 h). **d** Transwell assay of cells cotransfected with miR-10b-3p mimic and FOXO3 plasmids. **e** The expression of FOXO3 at the mRNA and protein level post siRNA silencing in KYSE150 cells. **f** Representative images and quantification of transwell assay after the transfection of FOXO3 siRNA into the KYSE150 cell lines. Each experiment was performed in triplicate. Data are presented as the mean value ± SD. ^●^, *P* < 0.05; ^●●^, *P* < 0.01

The authors sincerely apologize for the inconvenience caused to the readers.
