# Supplementary figures and images for: Promoter hypomethylation mediated upregulation of MicroRNA-10b-3p targets FOXO3 to promote the progression of esophageal squamous cell carcinoma (ESCC)
Source: J Exp Clin Cancer Res. 2018 Dec 4;37:301. doi: 10.1186/s13046-018-0966-1 (PMC6280546; doi:10.1186/s13046-018-0966-1)

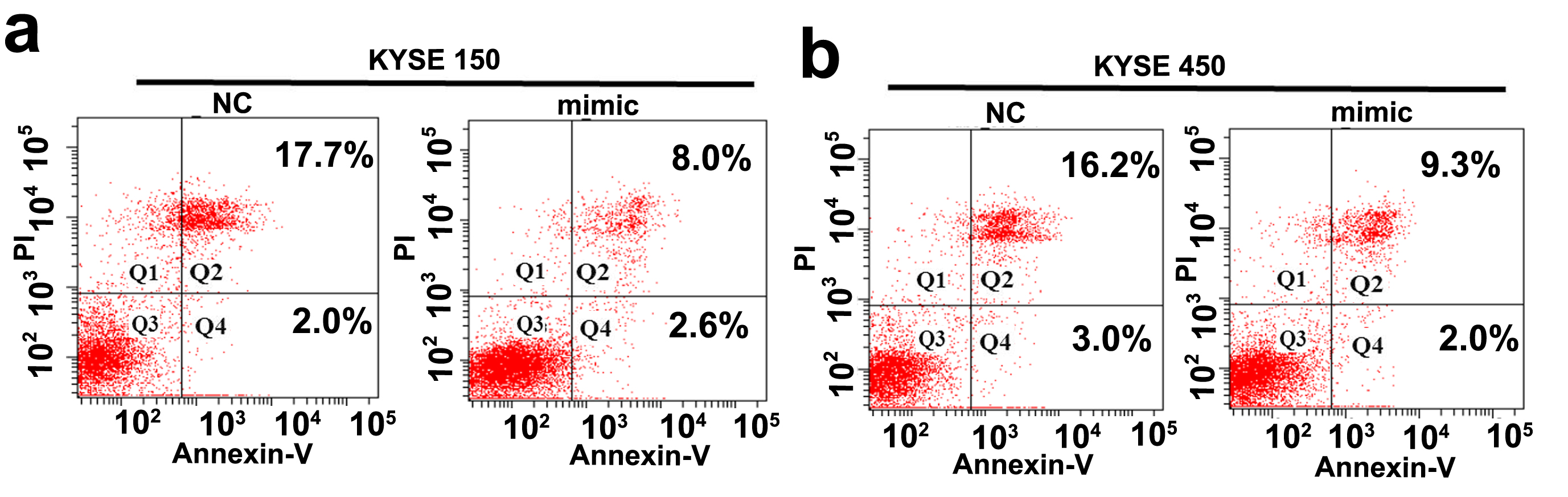

Supplement: Supplementary file 1 — Figrue S1. miR-10b-3p overexpression significantly inhibited cell apoptosis in ESCC cells. a miR-10b-3p reduced apoptosis in KYSE 150 cells. a miR-10b-3p reduced apoptosis in KYSE 450 cells. Each experiment was performed in triplicate. (JPG 890 kb) [file 13046_2018_966_MOESM1_ESM.jpg]

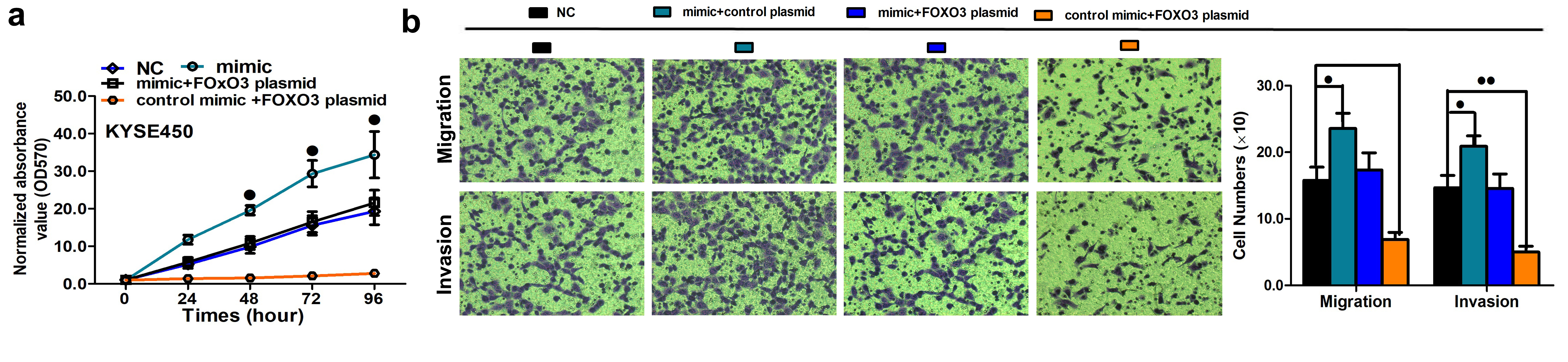

Supplement: Supplementary file 3 — Figure S2. A rescue assay was further performed to confirm that FOXO3 was the functional target of miR-10b-3p in KYSE450 cells. a The cell growth curve was measured by MTS cotransfected with miR-10b-3p mimic and FOXO3 plasmids in KYSE 450 cell lines, and the OD 570 was normalized to the star point (0 h). b Transwell assay of KYSE 450 cells with cotransfected with miR-10b-3p mimic and FOXO3 plasmids. (JPG 5228 kb) [file 13046_2018_966_MOESM3_ESM.jpg]

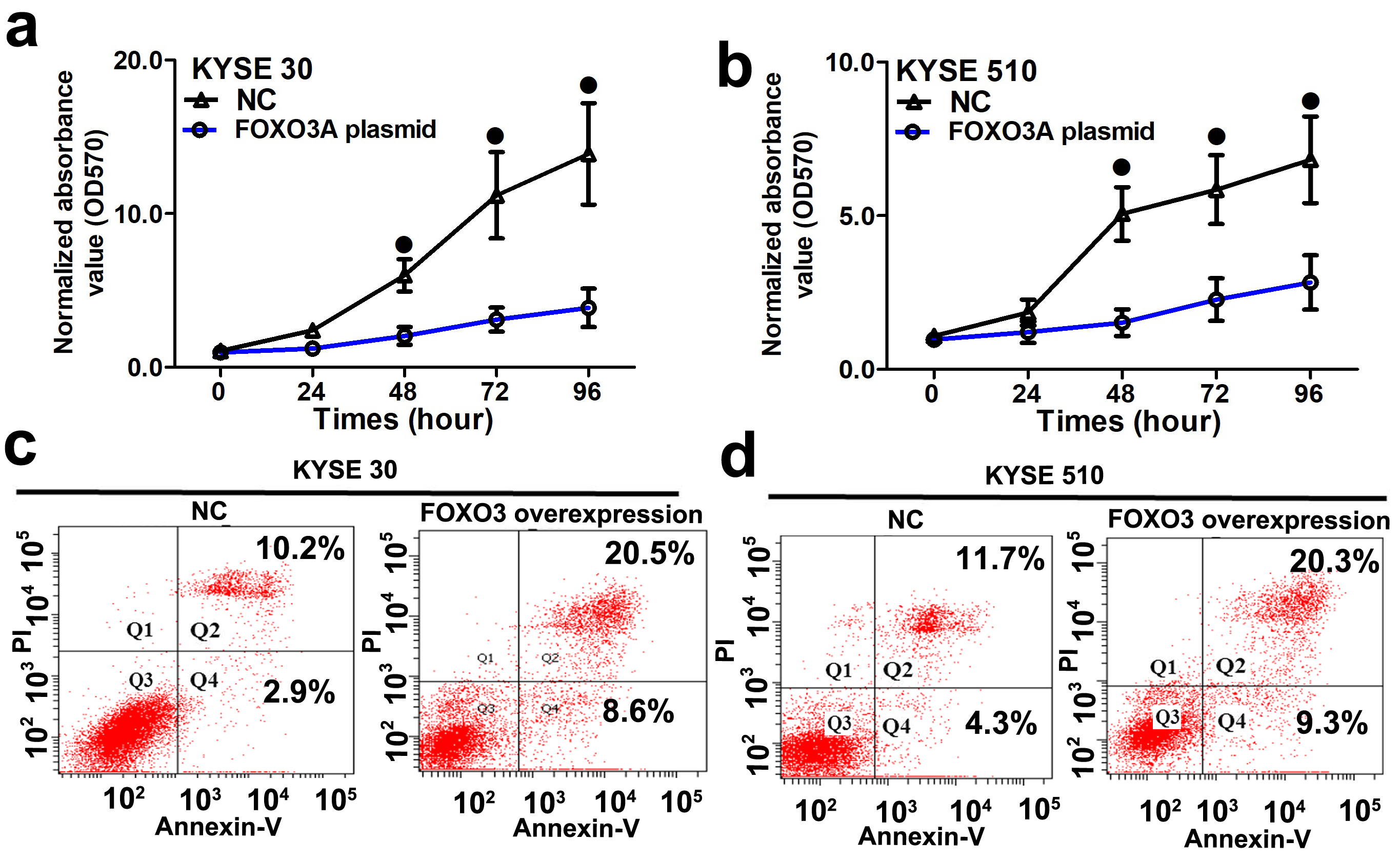

Supplement: Supplementary file 4 — Figure S3. FOXO3 plasmid overexpression significantly inhibited cell proliferation and promoted apoptosis in ESCC cells. a-b The cell growth curve was measured by MTS after transfection of the FOXO3 plasmid overexpression in KYSE30 and KYSE510 cell lines, and the OD 570 was normalized to the star point (0 h). c-d The cell apoptosis was measured by FACS analysis FOXO3 plasmid overexpression in KYSE 30 and KYSE 510 cell lines. Each experiment was performed in triplicate. (JPG 1648 kb) [file 13046_2018_966_MOESM4_ESM.jpg]
